# Supplementary material for: Human Milk Concentrations of Minerals, Essential and Toxic Trace Elements and Association with Selective Medical, Social, Demographic and Environmental Factors
Source: Nutrients. 2021 May 31;13(6):1885. doi: 10.3390/nu13061885 (PMC8228089; doi:10.3390/nu13061885)
Supplement: Supplementary file 1 [file nutrients-13-01885-s001.zip › Supplementary Table 1.pdf]

Supplementary Table 1: Configuration ICP-MS and ICP-OES spectrometers.

Abbreviations: Ag, silver; Al, aluminum; As, arsenic; Ba, barium; Be, beryllium; Ca, calcium; Cd, cadmium; Co, cobalt; Cr, chromium; Cs, cesium; Cu, copper; Fe, iron; Ga, gallium; Hg, mercury; I, iodine; ICP-MS, inductively coupled plasma mass spectrometry; ICP-

Component / Parameter	ICP-MS	ICP-OES
Nebulizer	Meihard	GemTip Flow Cross
Plasma gas flow	16.0 L/min	16.0 L/min
Auxiliary gas flow	1.2 L/min	0.5 L/min
Fog Rate	0.90 L/min	0.80 L/min
Sample Rate	400 µL/min	1.5 mL/min
Radio frequency power	1600 W	1600 W
Integration time	500 ms	5 s
Replicas per sample	3	5
Working mode	KED (Kinetic Energy Discrimination) 1 or 4 mL/min He	
Isotopes	^{7}Li, ^{9}Be,^{55}Mn, ^{63}Cu, ^{71}Ga, ^{85}Rb, ^{88}Sr, ^{98}Mo,^{107}Ag, ^{111}Cd, ^{133}Cs, ^{138}Ba, ^{195}Pt, ^{202}Hg, ^{205}TI, ^{208}Pb, ^{238}U<br>^{27}Al, ^{47}Ti, ^{51}V, ^{53}Cr, ^{57}Fe, ^{59}Co, ^{60}Ni, ^{66}Zn, ^{75}As, ^{78}Se, ^{118}Sn, ^{121}Sb, ^{127}I	Ca 317.93 nm<br>K 766.48 nm<br>Mg 285.21 nm<br>Na 589.584 nm<br>P 213.62 nm

OES, inductively coupled plasma optical emission spectrometry; K, potassium; Li, lithium; Mg, magnesium; Mn, manganese; Mo, molybdenum; Na, sodium; Ni, nickel; P, phosphorus; Pb, lead; Pt, platinum; Rb, rubidium; Sb, antimony; Se, selenium; Sn, tin; Sr, strontium; Ti, titanium; Tl, thallium; U, uranium; V, vanadium; Zn, zinc.
