# Supplementary material for: Human Milk Concentrations of Minerals, Essential and Toxic Trace Elements and Association with Selective Medical, Social, Demographic and Environmental Factors
Source: Nutrients. 2021 May 31;13(6):1885. doi: 10.3390/nu13061885 (PMC8228089; doi:10.3390/nu13061885)
Supplement: Supplementary file 1 [file nutrients-13-01885-s001.zip › Supplementary Table 2.pdf]

**Supplementary Table 2:** Concentrations of trace elements detected in human milk: comparison with range recommended by AAP[1]

| Element | Type of milk | AAP-limit range | Interval (95%) | | Lower range (%) | Upper range (%) | p-value | Element | AAP-limit range | Interval (95%) | | Lower range (%) | Above range (%) | p-value |
|---|---|---|---|---|---|---|---|---|---|---|---|---|---|---|
| | | | Lower | Upper | | | | | | Lower | Upper | | | |
| Minerals (mg/L) | | | | | | | | | | | | | | |
| Ca | TC | 200-300 | 233.43 | 257.29 | ***0*** | ***60*** | ***<0.001*** | Na | 120–250 | 106.14 | 231.61 | ***80*** | ***0*** | ***<0.001*** |
| | TI | | 250.92 | 290.27 | 0 | 13 | | | | 99.05 | 145.68 | 40 | 0 | |
| | TM | | 278.09 | 303.99 | 0 | 15 | | | | 110.18 | 139.68 | 40 | 0 | |
| | PM | | 280.36 | 310.17 | 0 | 15 | | | | 124.48 | 138.30 | 50 | 0 | |
| K | TC | 400–550 | 308.14 | 458.78 | 20 | 0 | >0.999 | P | 120–140 | 130.28 | 139.62 | 0 | 0 | >0.999 |
| | TI | | 332.84 | 467.28 | 40 | 0 | | | | 122.85 | 136.39 | 0 | 0 | |
| | TM | | 405.31 | 462.89 | 20 | 0 | | | | 121.67 | 135.10 | 10 | 0 | |
| | PM | | 352.92 | 494.38 | 30 | 0 | | | | 119.10 | 131.51 | 20 | 0 | |
| Mg | TC | 30–35 | 28.19 | 41.62 | 0 | 60 | >0.999 | | | | | | | |
| | TI | | 34.12 | 37.84 | 0 | 40 | | | | | | | | |
| | TM | | 36.21 | 40.17 | 0 | 20 | | | | | | | | |
| | PM | | 36.16 | 38.56 | 0 | 30 | | | | | | | | |
| Essential trace elements (µg/L) | | | | | | | | | | | | | | |
| Co | TC | 0–0.14 | 0.049 | 0.065 | 0 | 0 | >0.999 | Mn | 1–3 | 1.67 | 3.54 | 0 | 0 | >0.999 |
| | TI | | 0.037 | 0.067 | 0 | 0 | | | | 1.56 | 1.93 | 10 | 0 | |
| | TM | | 0.039 | 0.050 | 0 | 0 | | | | 1.44 | 1.92 | 0 | 0 | |
| | PM | | 0.049 | 0.056 | 0 | 0 | | | | 1.80 | 2.18 | 0 | 0 | |
| Cu | TC | 200-400 | 211.20 | 289.03 | 0 | 20 | 0.982 | Se | 10–30 | 9.90 | 11.73 | 50 | 0 | ***<0.001*** |
| | TI | | 236.02 | 302.29 | 0 | 10 | | | | 9.44 | 10.38 | 50 | 0 | |
| | TM | | 290.21 | 389.86 | 0 | 0 | | | | 8.28 | 9.47 | 60 | 0 | |
| | PM | | 251.03 | 279.63 | 0 | 0 | | | | 4.22 | 9.72 | **80** | 0 | |

| | | | | | | | | | | | | | | |
|---|---|---|---|---|---|---|---|---|---|---|---|---|---|---|
| Fe | TC | 300–900 | 162.82 | 211.32 | 0 | 0 | >0.999 | Zn | 1000– 3000 | 762.22 | 1248.20 | 10 | 0 | >0.999 |
| | TI | | 166.09 | 204.46 | 0 | 0 | | | | 797.25 | 1285.57 | 10 | 0 | |
| | TM | | 157.97 | 198.96 | 0 | 0 | | | | 1004.30 | 1471.22 | 10 | 0 | |
| | PM | | 119.71 | 190.15 | 0 | 0 | | | | 316.06 | 1401.85 | 0 | 0 | |
| I | TC | 40–250 | 94.96 | 122.3 | 0 | 0 | >0.999 | | | | | | | |
| | TI | | 109.37 | 134.52 | 0 | 0 | | | | | | | | |
| | TM | | 106.84 | 149.12 | 0 | 0 | | | | | | | | |
| | PM | | 84.57 | 105.79 | 0 | 0 | | | | | | | | |
| Toxic trace elements (μg/L) | | | | | | | | | | | | | | |
| Cs | TC | 0–5 | 2.84 | 8.12 | 0 | 10 | ***<0.001*** | Sr | 30–50 | 40.34 | 50.50 | 0 | 50 | ***<0.001*** |
| | TI | | 3.01 | 5.33 | 0 | 10 | | | | 34.33 | 42.03 | 0 | 30 | |
| | TM | | 2.71 | 3.55 | 0 | 10 | | | | 33.40 | 39.32 | 0 | 0 | |
| | PM | | 5.17 | 10.17 | 0 | ***70*** | | | | 42.78 | 55.95 | 0 | ***70*** | |

** Elements in bold and italic indicate the type of human milk statistically significant*

*Abbreviations: Ca, calcium; Co, cobalt; Cs, cesium; Cu, copper; Fe, iron; I, iodine; K, potassium; Mg, magnesium; Mn, manganese; Na, sodium; P, phosphorus; PM, preterm milk; Se, selenium; Sr, strontium; TC, full-term colostrum; TI, full-term intermediate milk; TM, full-term mature milk; Zn, zinc.*


1. American Academy of Pediatrics Committee on Nutrition. Trace elements. In: Pediatric nutrition, 8th, Kleinman RE, Greer FR (Eds), American Academy of Pediatrics, Itasca, IL 2019.
