# Supplementary material for: Human Milk Concentrations of Minerals, Essential and Toxic Trace Elements and Association with Selective Medical, Social, Demographic and Environmental Factors
Source: Nutrients. 2021 May 31;13(6):1885. doi: 10.3390/nu13061885 (PMC8228089; doi:10.3390/nu13061885)
Supplement: Supplementary file 1 [file nutrients-13-01885-s001.zip › Supplementary Table 3.pdf]

Supplementary Table 3: Concentrations of trace elements detected in infant milk formula: comparison with range recommended by ESPGHAN[1]

Element	Type milk	ESPGHAN limit range	Interval (95%)		Lower range (%)	Upper range (%)	p-value	Element	ESPGHAN limit range	Interval (95%)		Lower range (%)	Upper range (%)	p-value
			Lower	Upper						Lower	Upper			
Minerals (mg/L)														
Ca	SF	350–1050	345.14	494.72	10	0	>0.999	Na	130–390	131.72	162.40	40	0	>0.999
	CF		391.96	443.28	0	0				150.49	171.75	20	0	
	HF		322.49	537.90	10	0				113.57	267.72	10	0	
	PF		393	626	0	0				180	257	0	0	
K	SF	390–1100	448.92	497.21	20	0	>0.999	P	160–575	253.67	281.59	0	0	>0.999
	CF		436.12	451.62	30	0				261.15	288.77	0	0	
	HF		404.43	641.16	20	0				235.32	367	0	0	
	PF		432.28	662.71	0	0				271	381.59	0	0	
Mg	SF	35–110	46.46	54.46	0	0	>0.999							
	CF		40.36	65.88	0	0								
	HF		43.64	75.95	0	0								
	PF		62	73	0	0								
Essential trace elements (µg/L)														
Co	SF	0–2	0.17	0.33	0	0	>0.999	Mn	7–325	53.58	133.11	0	0	>0.999
	CF		0.16	0.34	0	0				34.27	80.43	0	0	
	HF		0.02	0.19	0	0				37.9	341.22	0	10	
	PF		0.34	1.08	0	0				31.37	79.02	0	0	
Cu	SF	225–520	337.25	428.81	0	0	>0.999	Se	660	15.95	21.21	0	0	>0.999
	CF		297.81	403.68	0	0				14.29	20.63	0	0	
	HF		314.82	479.84	0	0				16.10	25.94	0	0	
	PF		206.96	634.91	0	0				17.31	22.18	0	0	

Fe	SF	2000–10000	5369.12	6769.54	0	0	>0.999
	CF		8505.13	9346.36	0	0	
	HF		4939.29	9621.10	0	0	
	PF		2793.26	9814.03	0	0	
I	SF	65–325	114.05	152.00	0	0	>0.999
	CF		141.07	171.05	0	0	
	HF		131.96	148.17	0	0	
	PF		149.24	177.28	0	0	
Zn		3500 – 11000	4155.71	5139.35	10	0	0.872
			4015.30	5805.44	0	0	
			2970.98	7120.61	20	0	
			6179	7237	0	0	

Abbreviations*: Ca: calcium; CF, continuation formula; Co, cobalt; Cu, copper; ESPGHAN, European Society of Pediatric Gastroenterology and Nutrition; Fe, iron; HF; hydrolyzed formula; I, iodine; K, potassium; Mg, magnesium; Mn, manganese; Na, sodium; P, phosphorus; PF, preterm formula; Se, selenium; SF, starter formula; Zn, zinc.*

1. Hojsak I, Bronsky J, Campoy C et al; ESPGHAN Committee on Nutrition. Young Child Formula: A Position Paper by the ESPGHAN Committee on Nutrition. *J Pediatr Gastroenterol Nutr.* 2018; 66(1):177-185.
