# Supplementary material for: Human Milk Concentrations of Minerals, Essential and Toxic Trace Elements and Association with Selective Medical, Social, Demographic and Environmental Factors
Source: Nutrients. 2021 May 31;13(6):1885. doi: 10.3390/nu13061885 (PMC8228089; doi:10.3390/nu13061885)
Supplement: Supplementary file 1 [file nutrients-13-01885-s001.zip › Supplementary Table 4.pdf]

Supplementary table 4: Concentrations of trace elements detected in infant milk formula: comparison with manufacturer-reported concentrations

Element	Type milk	Manufacturer range	Interval (95%)		Lower range (%)	Upper range (%)	p-value	Element	Manufacturer range	Interval (95%)		Lower range (%)	Upper range (%)	p-value
			Lower	Upper						Lower	Upper			
Minerals (mg/L)														
Ca	SF	338–585	345.14	494.72	0	0	>0.999	Na	120–184	131.72	162.40	0	0	>0.999
	CF	405–650	391.96	443.28	0	0			159–184	150.49	171.75	0	0	
	HF	400–620	322.49	537.90	0	0			180–230	113.57	267.72	0	0	
	PF	500–620	393	626	0	0			215–254	180	257	0	10	
K	SF	440–569	448.92	497.21	0	0	>0.999	P	200–369	253.67	281.59	0	0	>0.999
	CF	440–554	436.12	451.62	0	0			243–327	261.15	288.77	0	0	
	HF	440–570	404.43	641.16	0	0			230–341	235.32	367	0	20	
	PF	500–590	432.28	662.71	0	0			300–365	271	381.59	0	20	
Mg	SF	31–51	46.46	54.46	0	20	>0.999							
	CF	33–60	40.36	65.88	0	30								
	HF	40–60	43.64	75.95	0	20								
	PF	40–70	62	73	0	10								
Essential trace elements (μg/L)														
Cu	SF	300–404	337.25	428.81	0	20	0.823	Se	11–21	15.95	21.21	0	10	>0.999
	CF	300–400	297.81	403.68	0	10			11–24	14.29	20.63	0	0	
	HF	305–410	314.82	479.84	0	30			7–21	16.10	25.94	0	20	
	PF	400–485	206.96	634.91	0	40			7–18	17.31	22.18	0	40	
Fe	SF	3000–6200	5369.12	6769.54	0	10	>0.999	Zn	3100–7300	4155.71	5139.35	0	0	>0.999
	CF	7000–10000	8505.13	9346.36	0	0			3500–8000	4015.30	5805.44	0	0	
	HF	3500–10000	4939.29	9621.10	0	0			3400–7600	2970.98	7120.61	0	0	
	PF	5300–7000	2793.26	9814.03	0	40			4000–8800	6179	7237	0	0	
I	SF	89–160	114.05	152.00	0	0	>0.999							
	CF	115–166	141.07	171.05	0	20								
	HF	89–145	131.96	148.17	0	20								

PF	89–180	149.24	177.28	0	0

Abbreviations*: Ca, calcium; CF, continuation formula; Cu, copper; Fe, iron; HF; hydrolyzed formula; I, iodine; K, potassium; Na, sodium; NS, not significant; P, phosphorus; PF, preterm formula; Se, selenium; SF, starter formula; Zn, zinc.*
